# Barriers to losing weight for women attending group visits in primary care: A qualitative exploration using in-depth interviews

**DOI:** 10.1080/13814788.2021.1998446

**Published:** 2021-11-15

**Authors:** Zeliha Yelda Özer, Sevgi Özcan, Gülşah Seydaoğlu, Hatice Kurdak

**Affiliations:** a Department of Family Medicine, Faculty of Medicine, Çukurova University, Adana, Turkey; b Department of Biostatistics, Faculty of Medicine, Çukurova University, Adana, Turkey

**Keywords:** Qualitative designs and methods**;** group medical visits, weight stigma, life trauma

## Abstract

**Background:**

Despite the accumulated evidence suggesting the positive aspects of using group visits in obesity, the number of qualitative studies that examine why and how the effects occur at an individual level is limited.

**Objectives:**

This qualitative study aimed to explore the experiences and perspectives of women who participated in group visits and had different weight loss outcomes in the programme.

**Method:**

Purposive maximum variation sampling was performed. Data collection and analysis were performed iteratively, and the data saturation method was used as a guideline for sample size. All participants who completed the group visits were approached, and finally, 20 individuals were included in the study. Semi-structured in-depth interviews were audio-recorded, transcribed verbatim, and analysed thematically using a phenomenological approach.

**Results:**

The mean age of the individuals was 38.5 ± 9.8 years, the education level ranged from incomplete-high school to university degree, and the weight changes were between +4.1% and −17.1%. Two main themes emerged from the thematic analysis revealing barriers: weight stigma (two sub-themes: internal and external stigma) and traumatic life events (three sub-themes: ‘loss of relatives,’ ‘childhood traumas,’ and ‘conflicting intimate partner relationships’).

**Conclusion:**

Considering the barriers to weight loss efforts in this study, these issues need to be explicitly investigated before and during the group visits in addition to weight loss practices and behavioural changes.

KEY MESSAGES

- Group visits are useful for the management of obesity. However, personal issues, such as weight stigma and life traumas, that cannot be shared during group visits may jeopardise weight loss efforts.
- When conducting group visits for obesity in primary care, healthcare professionals should consider that participants may have personal issues and may need individual consultation/referrals.

## Introduction

Lifestyle interventions form the basis for weight-loss programmes, and creating tailor-made options is essential to achieve the desired beneficial results [1]. Unfortunately, the time allocated to fulfil this task during conventional consultations in primary care lags to meet the demand due to the increased prevalence of obesity [2]. Group visits (GVs) can help healthcare professionals use their time efficiently and provide considerable benefits to patients with chronic conditions [3–5]. We previously conducted a study of family physician-led GVs for healthy lifestyle changes (HLCs) in women [6]. This previous study implemented a comprehensive intervention strategy comprising nutritional education, behavioural treatment techniques, problem-solving skills and self-monitoring studies. Most GV studies in obesity, including the one mentioned above, evaluated the outcomes through quantitative variables, and their average values were used to define success in showing the impact of the treatments. At the end of this study, women’s mean body weight decreased successfully. However, on an individual basis, it was observed that the participants had different weight loss ratios.

Despite the accumulated evidence suggesting the positive aspects of GVs in obesity, the number of qualitative studies that have examined why and how GVs work at an individual level is limited. A qualitative investigation of the various outcomes of the family physician-led GV-HLC studies may illuminate essential aspects of the problems and possible solutions. The present qualitative research aimed to explore the experiences and perspectives of women who participated in GVs and had different weight-loss outcomes in the programme using in-depth interviews (IDIs).

## Methods

### Study design

We used the phenomenological approach as a theoretically informed methodology for investigating obesity and weight loss efforts. While we evaluated IDIs iteratively, we observed patterns and analysed them thematically. The IDI method was chosen to explore the delicate issues [7]. The study was conducted and reported using the Consolidated Criteria for Reporting Qualitative Research (COREQ), and the checklist is presented in Supplementary Table 1 [8].

### Selection of study subjects

Purposive maximum variation sampling was performed. Of 30 participants who completed the GVs, seven could not be contacted and three could not participate in the study due to pregnancy, being out of town, or workload. Finally, 20 women were included in the study. Detailed descriptions of the participants’ weight characteristics categorised as regained (R), maintained (M), and negative cases (NC) are provided in Table 1. Data collection and analysis were performed iteratively, and the data saturation method was used as a guideline for sample size [7]. The IDIs started with the two R participants and the two NC and then mixed order. They were performed 6 months after the previous study was finalised. The study was approved by the Faculty of Medicine Ethics Committee of Çukurova University (ref: 40/06.03.2015). Informed written consent was obtained from all the participants.

**Table 1.** The characteristics of the participants.

Patient code (Enrolling order – Weight Maintenance on follow-up)	Age (years)	Education level/employment*	Relationship	Number of children	Weight change percentage (After the group visits)
P1–R	36	Master / Employed	Married	None	−4.0
P2–R	48	High school / Housewife	Married	2	−7.1
P3–NC	40	High school / Housewife	Married	2	+4.1
P4–NC	21	College / Student	Single	None	+1.3
P5–M	42	College / Employed	Married	2	−3.7
P6–R	36	College / Employed	Single	None	−12.6
P7–R	49	Master / Employed	Married	2	−12.7
P8–M	40	Master / Employed	Married	2	−7.3
P9–M	50	High school / Employed	Married	2	−10.9
P10–M	35	Master / Employed	Married	1	−10.5
P11–R	41	College / Employed	Married	2	−6.9
P12–R	22	College / Student	Single	None	−5.1
P13–M	41	Master / Employed	Married	1	−7.0
P14–M	42	Elementary / Housewife	Married	4	−13.4
P15–M	54	Elementary / Housewife	Widow	2	−12.8
P16–M	46	College / Employed	Married	2	−4.7
P17–R	43	College / Employed	Married	2	−16.2
P18–M	40	Master / Employed	Married	2	−17.1
P19–R	23	College / Student	Single	None	−11.5
P20–M	21	College / Student	Single	None	−3.3

*Education/occupation and relationship are blunted (given as a general description of confidentiality issues).

M: Maintained; defined as the participants who lost weight after the group visits and either lost more or did not gain weight at the end of the follow-up.

R: Regained; defined as the participants who lost weight after the group visits and regained weight at the end of the follow-up.

NC: Negative cases; defined as the participants who gained weight after the group visits and continued to gain weight at the end of the follow-up.

### Qualitative methods

A pilot interview in a mirrored room was conducted to reach a consensus on the methodology. The IDI questions were prepared based on trans-theoretical and cognitive-behavioural models over which GVs were founded [9]. Six semi-structured open-ended questions were asked without diversions using Socratic questioning when necessary (Supplementary Table 2). HK conducted the IDIs in her office, which is a familiar environment for the participants. A single password-protected computer under the responsibility of ZYÖ ensured data security and confidentiality of the audio records and transcripts. With the approval of the participants, ZYÖ attended the interviews to note visual cues.

### Data capture, coding and analysis

No re-recordings were taken, but one participant wanted to stop the recording before answering the fifth question. ZYÖ took explanatory notes about the context next to the text and discussed them with HK. After each IDI, HK and ZYÖ took notes and discussed their impressions of the interviews. In the subsequent triangulation stages, the researchers discussed what they felt, what the participants had meant, and the reflectivity of the researchers’ experiences. When determining the codes, they associated the notes with the participants’ comments within a hermeneutic cycle. The researchers read the transcripts independently, then re-read them together and discussed them until they reached a joint resolution [10]. After the analysis, main findings were shared with the participants individually and no one objected to the emerged themes.

## Results

All the participants (*N* = 20) were women aged 38.5 ± 9.8 years (mean ± standard deviation). The education level ranged from incomplete-high school to university degrees, and the weight changes ranged from +4.1% to −17.1% (Table 1). Two main themes emerged from the thematic analysis: weight stigma (two sub-themes) and traumatic life events (three sub-themes). A complete list of codes, categories, and themes developed through IDIs is presented in Supplementary Table 3.

### Weight stigma

#### External weight stigma

When analysing the impact of obesity on the participants’ lives and their weight loss efforts, how they addressed their weight problem and the themes of external stigma stood out. Eight participants declared that they experienced stigmatisation. The terms generally used in the community to imply obesity in the community negatively affected the participants. Five participants stated that healthcare professionals also made disturbing remarks.

*My feet slipped and I fell. My ankle hurt, so I went to the emergency room. The doctor in there umm… told me directly ‘They should not enrol the fats into this school’ in front of my friends. It was so humiliating. After that event, I went to the market and bought lots of chocolate and ate; this may be the reason why I failed. (P4–NC)*

*So, the word obesity means that you are in danger; the signs are blinking; it scares you; everything is over; there is no cure. It creates such anxiety. (P16–M)*

*For example, going to a wedding… You do not even want to dance. It is so embarrassing… When you are fat, you worry about everything you do and say. Well, like, at dinner, when I say this is tasty… Everything! I mean everything! No matter what it is, the conversation always turns around to weight. So, we are cautious about what we say and even what we joke about. (P1–R)*

One participant wanted to be invisible to avoid weight stigma and indirectly stated that she was introverted.

*She (an obese person) is probably an introvert and wants to stay in the background. I felt like that… wanted to hide away, be overlooked so that nobody would see me. I have even stopped looking after my hair just to avoid getting attention. (P4–NC)*

Eight participants stated that their family and social circles' intervention put pressure on them and adversely affected their efforts when trying to overcome their weight problems.

*How is it going? What did you do? These questions bug me. It is also because I fear failure! (P1–R)*

Seven participants perceived obesity as being caused by being female and linked the problem to the role of motherhood. They emphasised that cooking is seen as a woman's duty in Turkish culture and that mothers have a habit of finishing their children's leftovers.

*Kids were leaving food on their plate, and I did not want to throw it away, so I was eating even though I did not want to. (P5–M)*

Gender discrimination appears to affect both internal and external stigmatisation.

*Umm, I think women have more weight obsession… Having a figure that is liked is important. If she is married, ‘will my husband like me or cheat on me?’ If she is single, ‘what do guys think?’ ‘Does that suit look good on me?’ However, men do not have anxiety. If there is no health problem, they do not care about their weight. (P18–M)*

*Women are generally expected to be more well-groomed and aesthetically pleasing… perhaps it comes from the common consciousness of our society… (P8–M)*

##### Internal weight stigma

Numerous sabotaging automatic thoughts concerning weight problems emerged from the codes and categories of obesity. These thoughts seem to lead to dysfunctional coping behaviours. One of the striking findings was that obesity caused the participants to carry a persona to gain social acceptance.

*It is as if we need to be more understanding toward them to be accepted in a circle of friends. (P4–NC)*

One of the participants stated that the obese individuals she was familiar with pretended to be happy to hide their weight problems.

*All fat people are always happy… or are they? Do they just seem this way? I wonder if they just seem happy to hide their weight problems. (P19–R)*

The participants' thoughts and emotions about obese people were mostly negative and involved humiliation about obesity.

*‘They do not look good in what they wear.’ ‘I am ashamed of my body.’ ‘If I voice my opinion, people will eventually criticise my weight.’ ‘People look at obese people differently, even if they eat normal portions.’ ‘Nobody pays attention to what I say because I am obese.’ ‘I like to stay in the background because of my weight.’ ‘I pity them.’*

A participant described how she isolated herself when examining how her beliefs about obesity affected her behaviour. She stigmatises herself for her weight, isolates herself socially and restricts her relationships with others.

*I would not deem myself worthy of anything because I was fat. I would not do anything by myself. When I needed to do something on my own, I would ask a friend to come with me. This is due to a lack of self-confidence. Thinking that people would insult me… (P4–NC)*

The overstatement in one of the subject’s expressions relating to obesity was remarkable.

*I think they are very ignorant or weak. Well, they pursue happiness just by eating, like an animal, well, it is a bit rude but they are primitive. And some people, how should I say? It's a bit of slang, but they are losers! (P18–M)*

The participants’ negative beliefs about being obese and their dichotomous thinking drove them to dysfunctional coping behaviours and a vicious circle.

*It is unfair, my sister-in-law always eats but does not gain weight but I easily do. As I try to prevent it… I decide that I am going on a diet on Monday. Then it is Monday, and if I eat something that I was not supposed to, then I start to overeat. Then comes Tuesday… Well, I eat even more… (P14–M).*

*I was saying to myself, ‘Tomorrow I am going on a diet,’ but I would go somewhere the next day and forget my promise. Craassssh… Rock bottom… That idea in my head of ‘For crying out loud, nothing that I try to do ever works out,’ ‘Well, I might as well go the whole hog now.’ (P1–R)*

The codes and categories that arose from the participants’ thoughts about normal-weighted people revealed the subtheme of ‘the glorification of being slim’.

*They are very self-confident. They can present themselves very well in any environment. They can behave very comfortably. (P1–R)*

*I think how lucky she is, so lucky,…probably overeats but does not gain weight (P2–R)*

*I think they are always delighted. So happy, confident, cool… Life revolves around them. (P4–NC)*

### Traumatic life events

Thirteen participants talked about traumatic events in their lives. Twelve of them associated these with weight problems. The sub-themes were ‘loss of relatives’, childhood traumas’ and ‘conflicting intimate partner relationships.’

Two participants stated that the loss of relatives affected their eating behaviours.

*I always remember losing my cousin… she died at a very young age, I do not know how long I will live, so let me die eating. That is my way of thinking! (P1–R)*

*Losing my husband was significant grief. Losing my father a few years later was a different type of pain. When they were gone, out of distress, I would turn to food. (P14–M)*

One participant said that her grandparents raised her until she was 12 after her family went to work abroad. Her weight problems appeared after she moved in with her parents.

*It was tough for me to get used to my parents, so hard… (Crying) Thinking… how I had a happy childhood before. I do not remember overeating ever before. But after I went there, I started overeating. When something worried me, I would open the fridge and start eating. (P2–R)*

A participant was the oldest of the four siblings and felt that her mother did not take care of her well because she was inexperienced; therefore, she filled that gap by eating. This participant related her childhood traumas to her eating behaviour, which she interpreted as a milestone in life.

*In my family, well, how can I put it… I did not get much love. I mean, my mother is distant; she never hugged or kissed me. I fill this gap by eating. See, you fight with your husband, you feel lonely, so you eat something. Something bad happens in the office, you turn into your shell and eat. (P11–R)*

Another participant explained that she was sent to boarding school during her childhood and she replaced her feeling of unworthiness from bad dormitory conditions and the stress of being separated from her family with high-calorie fast food, the easiest thing to reach.

*I think this sense of unworthiness comes from those times… (voice trembling). This feeling prevents you from loving and caring for yourself… You need something to make you happy and to keep you alive. Without it, you try to make yourself happy with the food. (P13–M)*

A participant had a conflicting, intimate relationship. She was subjected to verbal violence from her boyfriend about her weight.

*I used to look beautiful in anything I wore before. He suppressed me so much that I felt hideous. We were together for five years… (crying). I was just sleeping and eating a lot at that time… I moved the bed in front of the TV and brought the food there. (P6–R)*

Although one participant lost weight in the first 2 months of the GVs, she completed the programme and gained weight. She said she almost divorced her husband because of another woman a few years ago yet decided to stay together for ‘the good of their children.’ When discussing the reasons for not conducting HLC, she stated that she felt more attractive when losing weight in the first few months. She thought it was like rewarding her husband.

*I was the one who was cheated on; I had to watch my weight and get thin again as if I was the guilty one. It is like trying to make him desire me again or offering myself to him. This idea made me feel like I was rewarding him, so I gave up trying to lose weight. (P3–NC)*

## Discussion

### Main findings

This study explored various weight-loss ratios and the two negative cases from the cognitive-behavioural perspective through in-depth interviews with 20 women who attended group visits. The aim was to understand each participant’s unique experience of obesity and weight loss phenomena. Two main themes emerged regarding the struggles of losing weight in group visits. Weight stigma and traumatic life events were significant factors affecting participants’ lives, regardless of their achievement in losing or maintaining weight.

### Comparison with the existing literature

Weight stigma is defined as negative attitudes and beliefs manifested in stereotypes, rejection, and prejudice against obese individuals [11,12]. Social, political and interpersonal stigmas are external forms that play an essential role in forming and maintaining internal stigma, reflecting negative social attitudes and beliefs towards obese people. Weight stigmatisation also manifests itself in the self-criticism of obese individuals and reveals how quickly the individual accepts negative stereotypes and attitudes towards him/herself [13].

The participants’ feelings and thoughts towards obese individuals were predominantly negative. When a small number of positive expressions were examined, it was observed that they were not authentic. The ‘Happy/cheerful fat person’ stereotype could be assumed to feel accepted under society’s imposition of the ideal female and external stigmatisation. Carr and Friedman reported that satisfaction and self-acceptance levels were lower among obese people than normal-weight people [14]. This difference may be related to the perception that they will be subject to discrimination because of obesity. Fennell and Jenkins emphasised the interaction between individuals’ beliefs about themselves and their opinions about how others evaluate them. Obese individuals combine the idea ‘I am weak as I cannot maintain my weight’ with the belief ‘People will think that I am not attractive because I am obese and do not have self-discipline’. This perception can have a combined effect on the self-esteem of the individual [15]. Experimental studies also show that stigma increases gorging, decreases exercise and dieting and prolongs obesity [16–18].

The fact that obesity leads to thoughts of despair, such as ‘everything is over’, ‘there is no solution’, should be considered, as it is an automatic thought that will prevent behavioural changes. Significantly, the participant who was subject to offensive behaviour from healthcare professionals due to her obesity displayed binge-eating behaviours after the incident. Physicians may have implicit or explicit prejudices and beliefs regarding individuals with weight problems [19]. Significant evidence suggests that biases towards obesity lead to adverse outcomes in emotional functioning, personal relationships, educational achievements, employment and health [20].

The participants also stated that the gender-based division of labour also influenced their weight. Cooking is a woman’s job and wasting food is considered a sin in Turkish society. Therefore, it is common for a mother to consume her children’s leftovers but there is no relevant scientific work on this topic. Although our study did not specifically address this subject, findings emerged regarding obesity, gender discrimination and gender-based labour division. The fact that the researchers and participants were all women may have promoted this theme to emerge.

According to role theory, men and women have different lifestyles dictated by their life roles that affect their health differently. Women’s domestic roles involve raising children and doing household chores, they do not have any spare time for exercise. Cooking is an integral part of a woman’s routine, and snacks increase their daily calorie intake [21]. The most crucial point is that women are subjected to this stigmatisation more than men, even at lower weights [22].

Beck has identified negative thoughts (rationalisations, underestimation of consequences, self-deluding thinking, arbitrary rules, mind-reading and exaggeration) that prevented dieters from reaching and maintaining their desired weight [23]. The participants’ automatic thoughts may also lead to internal stigmatisation, behavioural restriction, efforts to be accepted, and their entry into a vicious circle. It was observed that the participants entered a vicious circle by wrongfully thinking, ‘Everything I do is pointless’ and ‘I might as well go the whole hog’. It has been shown that dichotomous thinking is an essential indicator of weight regain [24,25].

Although positive and negative life milestones were emphatically requested during the interview, most participants spoke about traumatic life events as milestones associated with obesity and weight loss efforts. Traumatic life events seem to induce responses between the two extremes of food denial and emotional eating [26–28]. Adverse childhood experiences and post-traumatic stress disorder have been associated with obesity [27–28]. Interestingly, there were no participants in this group who presented with food denial.

In a phenomenological study of the marital quality of couples living with obesity, some couples described obesity as a third person who sometimes brought comfort and intimacy and sometimes pain and estrangement to the relationship. It was also observed that sometimes one of the partners in the triangle dealt with the partner by taking sides with weight, thus decreasing physical and emotional intimacy. Ledyard and Morrison emphasised that these couples were perhaps subconsciously pleased with the lack of physical and emotional intimacy caused by being overweight [29]. As for the other negative case in our study, the weight gain after the first months of weight loss was an incident of cheating and an unresolved conflict with a spouse. The participant, who thought that she ‘did not look attractive’ and did not want to ‘reward’ her husband by losing weight, subconsciously preferred to stay away from any intimacy by relying upon the third person, i.e. obesity in the marriage. Internal stigmatisation, which appears with the internalisation of the judgement ‘overweight people are not attractive’, can be mentioned in addition to the dysfunctional relationship.

We investigated patient satisfaction with group visits at the end of the previous study [8]. Most of the participants declared that they would participate in group visits in the future and recommend group visits to others. In another mixed-method study, we investigated patients’ opinions and suggestions about group visits [30]. Issues such as the inability of some participants to adequately express their problems, the fact that participants experienced comparison and discrimination among themselves also came to the fore.

### Strengths and limitations

This study allowed the participants to explain their stories and unique perspectives. The fact that all the researchers were female may have led to a unilateral interpretation of some findings. The researchers were of the same gender as the participants, making it easier to understand and interpret the information.

A 6-month gap between the two studies may have resulted in recall bias. However, the time scale the participants may have been experiencing obesity is often far longer than this period. Group visit participants were recruited from applicants through advertisements posted in the city, university campus, and dormitories. Since group visits are a new method, they may have attracted women who had tried different methods before and failed. Therefore, the fact that there were too many negative thoughts identified in this study should be interpreted cautiously. Although these factors restrict the generalisability of the study’s findings, the purpose of qualitative studies is not to reflect the findings on the general population but to present an in-depth view of a particular subject group’s meanings and relative realities.

### Practical implications and future perspectives

One of the most critical findings in the present study is that regardless of weight loss, weight stigma negatively affected and constrained the participants’ lives and weight loss efforts. The experience of each individual with obesity was personal and unique. Therefore, women’s experiences, attitudes, and beliefs should be valued and considered when planning weight-loss interventions. The prevalence of weight stigma in society among health professionals and the density of its effects is a significant obstacle to patients and professionals fighting against obesity. Therefore, in addition to HLC-related studies, focussing on the possible effects of weight stigma on people’s self-actualisation in a real-life context may be more efficient than simply focussing on weight goals. It should be kept in mind that the meaning ascribed to weight loss, the potential impacts of the perception of weight loss between intimate partners and relationships with unresolved conflicts could all have an adverse role in the patients’ efforts to lose weight.

### Suggestion for future research

Investigating different group visit models (personalised sessions at the beginning, in the middle, and at the end of the group visits) for different cultures can be valuable in improving the method. The assumption that the results may be valid for similar problems is open to verification by quantitative or mixed studies.

## Conclusion

Considering the psychosocial, cultural and existential factors associated with the underlying context of body weight, the focus of obesity management can positively affect adherence and coherence, as well as behavioural changes and programme output. Personal issues, such as weight stigma and trauma that cannot be noticed during group visits, may jeopardise success. Healthcare professionals should keep in mind that the participants may have unresolved personal issues and may need an individual consultation.

## Supplementary Material

Supplementary Table 3.

Supplementary Table 2.

Supplementary Table 1.

## Data Availability

All interviews are available at the Family Medicine Department of Çukurova University, Turkey, from Dr. Yelda Özer.

## References

[1] Schutz DD, Busetto L, Dicker D, et al. European practical and patient-centred guidelines for adult obesity management in primary care. Obes Facts. 2019;12(1):40–66. 30673677 10.1159/000496183 PMC6465693

[2] Irving G, Neves AL, Dambha-Miller H, et al. International variations in primary care physician consultation time: a systematic review of 67 countries. BMJ Open. 2017;7(10):e017902. 10.1136/bmjopen-2017-017902 PMC5695512 29118053

[3] Edelman D, McDuffie JR, Oddone E, et al. Shared medical appointments for chronic medical conditions: a systematic review. VA Evidence-based Synthesis Program Reports [Internet]. 2012. [cited 2021 October 21]. Available from: http://www.ncbi.nlm.nih.gov/books/NBK99785/. 22916369

[4] Booth A, Cantrell A, Preston L, et al. What is the evidence for the effectiveness, appropriateness and feasibility of group clinics for patients with chronic conditions? A systematic review. Health Serv Deliv Res. 2015;3(46):1–194. 26726695

[5] Wadsworth KH, Archibald TG, Payne AE, et al. Shared medical appointments and patient-centered experience: a mixed-methods systematic review. BMC Fam Pract. 2019;20(1):1–13. 31286876 10.1186/s12875-019-0972-1 PMC6615093

[6] Tunay M, Kurdak H, Özcan S, et al. Family physician-led group visits for lifestyle modification in women with weight problems: a pilot intervention and follow-up study. Obes Facts. 2018;11(1):1–14. 10.1159/000486133 PMC5869599 29402785

[7] Ritchie J, Lewis J. Qualitative research practice: a guide for social science students and researchers. London (UK): SAGE Publication; 2014.

[8] Tong A, Sainsbury P, Craig J. Consolidated criteria for reporting qualitative research (COREQ): a 32-item checklist for interviews and focus groups. Int J Qual Health C. 2007;19(6):349–357. 10.1093/intqhc/mzm042 17872937

[9] Beck AT, Rush AJ, Shaw BF, et al. Cognitive therapy of depression. New York (NY): The Guilford Press; 1979.

[10] Miles MB, Huberman MA, Saldana J. Chapter 11: drawing and verifying conclusions. In Qualitative data analysis: a methods sourcebook. Los Angeles (CA): SAGE Publication; 2014. p. 275–322.

[11] Puhl RM, Moss-Racusin CA, Schwartz MB, et al. Weight stigmatisation and bias reduction: perspectives of overweight and obese adults. Health Educ Res. 2007;23(2):347–358. 17884836 10.1093/her/cym052

[12] Latner JD, O'Brien KS, Durso LE, et al. Weighing obesity stigma: the relative strength of different forms of bias. Int J Obes. 2008;32(7):1145–1152. 10.1038/ijo.2008.53 18414421

[13] Durso LE, Latner JD, White MA, et al. Internalized weight bias in obese patients with binge eating disorder: associations with eating disturbances and psychological functioning. Int J Eat Disord. 2012;45(3):423–427. 21717488 10.1002/eat.20933 PMC3184343

[14] Carr D, Friedman MA. Is obesity stigmatising? bodyweight, perceived discrimination, and psychological well-being in the United States. J Health Soc Behav. 2005;46(3):244–259. 16259147 10.1177/002214650504600303

[15] Fennell M, Jenkins H, et al. Low self-esteem. In: Bennett-Levy J, Butler G, Fennell M. editors. Oxford guide to behavioural experiments in cognitive therapy. Oxford: Oxford University Press; 2015. p. 413–430.

[16] Major B, Eliezer D, Rieck H. The psychological weight of weight stigma. Soc Psychol Pers Sci. 2012;3(6):651–658.

[17] Nolan LJ, Eshleman A. Paved with good intentions: paradoxical eating responses to weight stigma. Appetite. 2016;102:15–24. 26802721 10.1016/j.appet.2016.01.027

[18] Latner JD, Wilson GT, Jackson ML, et al. Greater history of weight-related stigmatising experience is associated with greater weight loss in obesity treatment. J Health Psychol. 2009;14(2):190–199. 19237486 10.1177/1359105308100203

[19] Sabin JA, Marini M, Nosek BA. Implicit and explicit anti-fat bias among a large sample of medical doctors by BMI, race/ethnicity and gender. PLoS One. 2012;7(11):e48448. 23144885 10.1371/journal.pone.0048448 PMC3492331

[20] Kyle TK, Puhl RM. Putting people first in obesity. Obesity (Silver Spring). 2014;22(5):1211. 24616446 10.1002/oby.20727

[21] Batnitzky A. Obesity and household roles: gender and social class in Morocco. Sociol Health Ill. 2008;30(3):445–462. 10.1111/j.1467-9566.2007.01067.x 18373507

[22] Boswell RG, White MA. Gender differences in weight bias internalisation and eating pathology in overweight individuals. Adv Eat Disord. 2015;3(3):259–268. 27042387 10.1080/21662630.2015.1047881 PMC4814168

[23] Beck JS, Deborah BB. The diet trap solution: train your brain to lose weight and keep it off for good. New York (NY): Hay House UK; 2015.

[24] Byrne S, Cooper Z, Fairburn C. Weight maintenance and relapse in obesity: a qualitative study. Int J Obes Relat Metab Disord. 2003;27(8):955–962. 12861237 10.1038/sj.ijo.0802305

[25] Rogerson D, Soltani H, Copeland R. The weight-loss experience: a qualitative exploration. BMC Public Health. 2016;16(1):1–12. 27142984 10.1186/s12889-016-3045-6 PMC4855339

[26] Zipfel S, Giel KE, Bulik CM, et al. Anorexia nervosa: aetiology, assessment, and treatment. Lancet Psychiat. 2015;2(12):1099–1111. 10.1016/S2215-0366(15)00356-9 26514083

[27] Wiss DA, Brewerton TD. Adverse childhood experiences and adult obesity: a systematic review of plausible mechanisms and Meta-analysis of cross-sectional studies. Physiol Behav. 2020;223:112964. 32479804 10.1016/j.physbeh.2020.112964

[28] Dorflinger LM, Masheb RM. PTSD is associated with emotional eating among veterans seeking treatment for overweight/obesity. Eat Behav. 2018;31:8–11. 30048898 10.1016/j.eatbeh.2018.07.005

[29] Ledyard ML, Morrison NC. The meaning of weight in marriage: a phenomenological investigation of relational factors involved in obesity. Journal of Couple & Relationship Therapy. 2008;7(3):230–247.

[30] Özer ZY, Kurdak H, Özcan S. Evaluation of the group visits method used in obesity management. TJFM&PC. 2018;12(4):288–300.

