# Supplementary material for: Barriers to losing weight for women attending group visits in primary care: A qualitative exploration using in-depth interviews
Source: Eur J Gen Pract. 2021 Nov 15;27(1):331–8. doi: 10.1080/13814788.2021.1998446 (PMC8604469; doi:10.1080/13814788.2021.1998446)
Supplement: Supplementary Table 3. [file IGEN_A_1998446_SM7496.docx]

Supplementary Table 3. A list of codes, categories, subthemes, and themes developed from interview data.

| **THEMES** | **SUBTHEMES** | **CATEGORIES** | **CODES** |
| --- | --- | --- | --- |
| **1. WEIGHT STIGMA** | **1. INTERNAL STIGMA**  **Striving/Pretending for social acceptance** | **Thoughts-regarding OWP/OP** | Complacent  Compassionate  Full of love*  Cheerful*  Happy*  Sympathetic* |
|  | **The humiliation of being overweight or obese** | **Emotions-towards OWP/OP** | Pitiful  Unattractive  Shame  Irritable  Quick-tempered  Angry  Terrified  Regretful  Confused  Bored  Insecure  Restless  Worried  Sensitive  Pessimistic  Sad /Unhappy  Introvert  Desperate  Alone/Isolated  Helpless  Insufficient  Weak-minded  Unsuccessful  Disgusting  Distressed  Restricted  Hateful (to herself)  Feeling under pressure (because of the perception that women must look better)  Undecided |
|  | **Belief in weight problems and dysfunctional coping strategies** | **Participants' thoughts on the causes of obesity** | Genetic predisposition  Bad luck  Personal problems  Inability to control oneself  Weakness of will  Unconscious eating/living  Ignorance, primitiveness  Injustice |
|  | **The glorification of being slim** | **Participants' thoughts about being slim** | Self-confident  Self-controlled  Independent  Feeling good  Lucky to have a fast metabolism  Charming  Brave  Successful  Looks good in clothes  Sexy, attractive  Energetic  Their lives go easy  Move comfortably  Wear what they want  The world revolves around them |
|  | **2. EXTERNAL STIGMA**  **Influence of the social environment** | **The thoughts/behaviours of the participants in their environment about obese or overweight individuals** | Restricted in most of the things  People disapprove them  People make fun of them  People look at them oddly even if they eat normal portions  Nobody pays attention to what they say  Negative attitudes and appeals of healthcare professionals  Bullied by family members  Bullied by colleagues |
| **2. TRAUMATIC LIFE EVENTS** | **Cultural factors** | **The effect of their behaviour on being obese or overweight** | The woman's duty to cook  The woman's duty to prepare the table  Eating leftover food while clearing the table |
|  | **Gender discrimination** | **Participants' opinions on female and male OWP/OP** | Women's obesity is more noticeable than men's  Women must be slim  Obesity is a bigger problem in women  Women must be more elegant  Women must be aesthetic |
|  | 1. **DEATH OF RELATIVES** | **Reasons to stop striving for weight control** | Death  Mourning  Mortality |
|  | 1. **CHILDHOOD TRAUMAS (VIOLENCE, NEGLECT)** | **Trying to make yourself happy by eating** | Going to boarding school under challenging conditions and food comfort  Violence from father and food comfort  Not getting the love, affection, or attention expected from the family |
|  | **3. CONFLICTING INTIMATE RELATIONSHIP** | **The effect of close relationships on obesity** | Partner abuse with weight stigma  Being cheated on |

OWP: overweight people, OP: obese people, *to be accepted or approved
