# Supplementary material for: Barriers to losing weight for women attending group visits in primary care: A qualitative exploration using in-depth interviews
Source: Eur J Gen Pract. 2021 Nov 15;27(1):331–8. doi: 10.1080/13814788.2021.1998446 (PMC8604469; doi:10.1080/13814788.2021.1998446)
Supplement: Supplementary Table 2. [file IGEN_A_1998446_SM7495.docx]

Supplementary Table 2. Semi-structured questions and inquiry rationale for in-depth interviews.

| **Objective** | **Question** | **Prompts & Probes** |
| --- | --- | --- |
| **Understand their views on being overweight and normal weight*** | 1. What are your thoughts on normal-weight individuals? 2. What are your emotions regarding them? 3. What are your thoughts on overweight/obese individuals? 4. What are your emotions regarding them? | Why do you think it could be like this?  Could you explain a bit more?  Does this affect your behaviour?  How does this affect your lifestyle modification efforts?  What effect would this have?  Can you give an example? |
| **Elicit the participant’s bio-psychosocial, existential and cultural status**** | 1. Could you tell me some of the important events that influenced you positively or negatively through your life? |  |
| **Prevent the doorknob phenomenon and facilitate disclosing sensitive subjects** | 1. Would you like to add anything regarding the subject? |  |

* Designed indirectly to pass from general to personal so that she could express her sensitive feelings and thoughts comfortably.

** Examine the subject in an integrated way based on the principles of family medicine
