# Supplementary material for: Barriers to losing weight for women attending group visits in primary care: A qualitative exploration using in-depth interviews
Source: Eur J Gen Pract. 2021 Nov 15;27(1):331–8. doi: 10.1080/13814788.2021.1998446 (PMC8604469; doi:10.1080/13814788.2021.1998446)
Supplement: Supplementary Table 1. [file IGEN_A_1998446_SM7494.docx]

Supplementary Table 1. COREQ 32-item checklist [8]

| Topic and Item No. | Guide Questions/Description | Explanations | Page No. |
| --- | --- | --- | --- |
| **Domain 1: research team and reflexivity** | | |  |
| Personal Characteristics |  |  |  |
| 1. Interviewer/facilitator | Which author/s conducted the interview or focus group? | Hatice Kurdak (HK) |  |
| 2. Credentials | What were the researcher’s credentials? e.g. PhD, MD | Hatice Kurdak, Family Physician, MD  Z.Yelda Özer, Family Physician, MD  Sevgi Özcan, Family Physician, MD  Gülşah Seydaoğlu, Biostatistician, MD |  |
| 3. Occupation | What was their occupation at the time of the study? | Hatice Kurdak, Sevgi Özcan, and Gülşah Seydaoğlu were faculty; Z.Yelda Özer was  research assistant |  |
| 4. Gender | Was the researcher male or female? | Female |  |
| 5. Experience and training | What experience or training did the researcher have? | HK and SO have cognitive-behavioural therapy-based interview training and are working on lifestyle change as their chosen field of study. GS, working in the Biostatistics Department, is the author of a periodical feminist journal and has an undergraduate degree in sociology. |  |
| Relationship with participants | | |  |
| 6. Relationship established | Was a relationship established prior to study commencement? | HK became well acquainted with the participants as she conducted the group visits and individual interviews for the previous study in 2014 |  |
| 7. Participant knowledge of the interviewer | What did the participants know about the researcher? e.g., personal goals, reasons for doing the research | The purpose of the study was summarised verbally, and the informed consent form was provided to the participants |  |
| 8. Interviewer characteristics | What characteristics were reported about the interviewer/facilitator? e.g., Bias, assumptions, reasons, and interests in the research topic | Participants were familiar with HK from the previous weight loss intervention study and knew she is a family physician with an interest in healthy lifestyle change practices |  |
| **Domain 2: study design** | | |  |
| Theoretical framework |  |  |  |
| 9. Methodological orientation and  Theory | What methodological orientation was stated to underpin the study? e.g., grounded theory, discourse analysis, ethnography, phenomenology, content analysis | Phenomenology & Thematic analysis |  |
| Participant selection | | |  |
| 10. Sampling | How were participants selected? e.g., purposive, convenience, consecutive, snowball | Purposive sampling & maximum variance |  |
| 11. Method of approach | How were participants approached? e.g., face-to-face, telephone, mail, email | Face-to-face |  |
| 12. Sample size | How many participants were in the study? | 20 |  |
| 13. Non-participation | How many people refused to participate or dropped out? Reasons? | None |  |
| Setting | | |  |
| 14. Setting of data collection | Where was the data collected? e.g., home, clinic, workplace | Çukurova University, HK’s office |  |
| 15. Presence of non-participants | Was anyone else present besides the participants and researchers? | With the approval of the participants, ZYÖ attended the interviews to note down visual cues. |  |
| 16. Description of sample | What are the important characteristics of the sample? e.g., demographic data, date | Gender, age, education level/occupation, number of children, and weight change percentage after the intervention and follow-up period |  |
| Data collection | | |  |
| 17. Interview guide | Were questions, prompts, guides provided by the authors? Was it pilot tested? | Yes, six semi-structured open-ended questions were prepared to reflect on the previous programme's participants' personal experiences. The in-depth interview questions were pilot tested |  |
| 18. Repeat interviews | Were repeat interviews carried out? If yes, how many? | No |  |
| 19. Audio/visual recording | Did the research use audio or visual recording to collect the data? | Data were audio-recorded with a digital recorder. |  |
| 20. Field notes | Were field notes made during and/or after the interview or focus group? | Yes, during and after the interviews |  |
| 21. Duration | What was the duration of the interviews or focus group? | Ranged between 21-53 min (573 min total) |  |
| 22. Data saturation | Was data saturation discussed? | Yes |  |
| 23. Transcripts returned | Were transcripts returned to participants for comment and/or correction? | No |  |
| **Domain 3: analysis and findings** | | |  |
| Data analysis | |  |  |
| 24. Number of data coders | How many data coders coded the data? | 2 |  |
| 25. Description of the coding tree | Did authors provide a description of the coding tree? | Yes (Supplementary Table 3) |  |
| 26. Derivation of themes | Were themes identified in advance or derived from the data? | Themes were derived from the data. |  |
| 27. Software | What software, if applicable, was used to manage the data? | ZYÖ transcribed the data verbatim into Word documents and grouped them in tables. |  |
| 28. Participant checking | Did participants provide feedback on the findings? | Not the transcripts but the summary of the findings were shared with the participants |  |
| Reporting | | |  |
| 29. Quotations presented | Were participant quotations presented to illustrate the themes/findings? Was each quotation identified? e.g. participant number | Yes |  |
| 30. Data and findings consistent | Was there consistency between the data presented and the findings? | Yes |  |
| 31. Clarity of major themes | Were major themes clearly presented in the findings? | Yes |  |
| 32. Clarity of minor themes | Is there a description of diverse cases or discussion of minor themes? | Yes |  |

* COREQ: Consolidated criteria for reporting qualitative studies
